# Risk factor analysis and nomogram construction for predicting suicidal ideation in patients with cancer

**DOI:** 10.1186/s12888-022-03987-z

**Published:** 2022-05-24

**Authors:** Yuanyuan Luo, Qianlin Lai, Hong Huang, Jiahui Luo, Jingxia Miao, Rongrong Liao, Zhihui Yang, Lili Zhang

**Affiliations:** 1 School of Nursing, Southern Medical University, No. 1023 Sha Tai South road, Baiyun district, Guangzhou, 510515 China; 2 Department of Medical Oncology, Nanfang Hospital, Southern Medical University, No. 1838, North Guangzhou Avenue, Baiyun District, Guangzhou, 510515 China; 3 First Nursing Unit of Tumor Ward, Integrated Hospital of Traditional Chinese Medicine, Southern Medical University, No. 13, Pomegranate Gang Road, Haizhu District, Guangzhou, 510315 China

**Keywords:** Suicidal ideation, Risk factor, Nomogram, Cancer patients

## Abstract

**Background:**

Suicidal ideation in cancer patients is a critical challenge. At present, few studies focus on factors associated with suicidal ideation, and predictive models are still lacking. This study aimed at investigating the risk factors for suicidal ideation among cancer patients, and developed a predictive nomogram to screen high risk cancer patients for early prevention and intervention.

**Methods:**

A questionnaire survey was conducted among cancer patients between May 2021 and January 2022. The factors associated with suicidal ideation were used to construct a multivariate logistic regression model, which was visualized as a predictive nomogram to evaluate the risk of suicidal ideation. Areas under the curve, calibration plot, decision curve analysis, and internal and external validation were used to validate the discrimination, calibration and clinical usefulness of the model.

**Results:**

A total of 820 patients with cancer were recruited for this study and 213 (25.98%) developed suicidal ideation. Levels of demoralization, depression and cancer staging, marital status, residence, medical financial burden, and living condition were influence factors for suicidal ideation. Comparing nomogram with Self-rating Idea of Suicide Scale (SIOSS), the nomogram had a satisfactory discrimination ability with an AUC of 0.859 (95% CI: 0.827–0.890) and 0.818 (95% CI: 0.764–0.873) in the training and validation sets, respectively. The calibration plot and decision curve analysis revealed that this nomogram was in good fitness and could be beneficial in clinical applications.

**Conclusions:**

Suicidal ideation is common in cancer patients. Levels of demoralization, depression and cancer staging were independent predictors of suicidal ideation. The nomogram is an effective and simple tool for predictive suicidal ideation in cancer patients.

**Supplementary Information:**

The online version contains supplementary material available at 10.1186/s12888-022-03987-z.

## Background

Suicide is a devastating public health concern. The World Health Organization estimates that more than 800,000 people die by suicide every year, about one person every 40 s [1–3]. Any serious medical diagnosis may increase a patient's risk of suicide, of which cancer diagnosis is one of the most devastating events [4]. Cancer as a leading cause of death around the world, with 19.3 million new cancer cases and almost 10.0 million cancer deaths in 2020, and it will continue to grow [5]. As medicine progresses and updates the treatment, more and more cancer patients die of non-cancer causes [6]. But they also suffer from a great deal of physical and psychological stress. Cancer diagnosis causes mental breakdown, cancer treatments generate side effects, cancer stigma leads to social isolation, and cancer costs also leave a financial burden, all of which could push cancer patients at a high risk of suicide [7–10]. Due to the destructiveness and rapidity of suicide, suicide has become one of the main reasons behind the death rate affecting cancer patients [11, 12]. Therefore, cancer patients are 3 to 4 times more likely to commit suicide than other people [13]. Taken together, the combination of suicide and cancer is such a pernicious issue for cancer patients. In China, suicide accounts for over one-quarter to one-third of suicides worldwide, and it also the highest cancer proportion of all countries [14–16]. Additionally, as a result of traditional Chinese culture’s taboo and stigma toward suicide and cancer, the incidence of suicide in cancer patients may be even worse than reported.

The first step towards prevention of suicide is to identify cancer patients who are at high risk of suicide. Suicidal ideation (SI), often called suicidal thoughts or ideas, refers to having thoughts, ideas, or ruminations about the possibility of ending one's life [17, 18]. Suicide includes a process from SI to suicide attempt and behavior, SI is the most important sentinel manifestation in this process [19]. Therefore, screening SI can help identify patients who are at high risk of suicide. Previous studies have focused on identifying influence factors, such as age, gender, symptom burden, and time of cancer diagnosis [6, 20, 21], but these may not be applied in clinical practice directly. In addition, the emotional status of individuals was seldom covered. The risk factors associated with SI have not been adequately documented, and reliable prediction models are still lacking. Thus, the conundrum of SI in cancer patients urgently needs to be settled.

Nomograms have been widely accepted as reliable tools to predict individual risk in reported outcomes [22]. However, nomograms are rarely used to predict SI. This study investigated the prevalence and potential risk factors of SI among cancer patients in China, and developed a nomogram to predict SI in cancer patients, which may help medical staff quickly identify cancer patients with high risk of SI and prevent suicide tragedies.

## Methods

### Study population

The cancer patients were recruited from Nanfang Hospital and Integrated Hospital of Traditional Chinese Medicine, Southern Medical University from May 2021 to January 2022. The inclusion criteria were pathologically confirmed cancer (ICD-10 code C00-C97), age ≥ 18 years old, clear consciousness, and ability to speak or write. The exclusion criteria were presence of psychotic symptoms and received psychotropic medication within 2 weeks, and incomplete medical records.

According to different hospitals, patients were divided into a training set (560 patients of Nanfang Hospital, Southern Medical University) and a validation set (260 patients of Integrated Hospital of Traditional Chinese Medicine, Southern Medical University). The training set was used for both model development and internal validation, while the validation set was used for external validation of the model.

### Measures

Socio-demographic characteristics of cancer patients were obtained from the hospital’s electronic medical record system, including age, gender, type of cancer, cancer staging, level of education, religious belief, and marital status. Other information was obtained by asking patients about their residence, caretakers, income, living conditions, current employment status, and medical financial burden. The personal information of subjects was removed to provide privacy protection.

#### Self-rating Idea of Suicide Scale (SIOSS)

The SIOSS is a 26-item self-report questionnaire, which includes the despair factor, optimistic factor, sleep factor, and masking factor [23]. Each item has two options: “Yes” and “No”. The total score is the sum of the subscales, a total score ≥ 12 was deemed suicidal ideation occurred, and the higher the total score, the stronger the suicidal ideation. SIOSS is one of the widely used questionnaires to evaluate suicidal ideation in China, and reliability and validity of SIOSS are satisfactory [24–26]. The internal consistency reliability (Cronbach’s α coefficient) of the SIOSS in this study was 0.762.

#### Chinese version of Demoralization Scale II (DS-II-C)

The demoralization was assessed using the DS-II-C, which is a 16-item questionnaire and was translated from DS-II. DS-II-C covers two 8-item subscales (Meaning and Purpose and Distress and Coping Ability). Three-point Likert scale was used, zero means never, one means sometimes, two means often. Total scores ranging between 0 and 32 and the partition criterion are defined as between 25 and 75th percentile as the author suggested [27]. In this study ≤ 5 points (0-25th percentile) means low demoralization, 5–17 points (25th-75th percentile) means moderate demoralization, and ≥ 18 points (≥ 75th percentile) means high demoralization. The internal consistency reliability (Cronbach’s α coefficient) of the DS-II-C in this study was 0.925.

#### The Hospital Anxiety and Depression Scale (HADS)

The severity of depression and anxiety were assessed using the HADS, which includes two 7-item subscales (Anxiety and Depression) [28]. Each item has four options (0–3), which represents a degree from not at all to very much. The total score for each subscale is the sum of seven entries. The normal range is 0 to 7 points; 8 to 10 is mild, 11 to 14 is moderate, and 15 to 21 is severe anxiety or depression. The internal consistency reliability (Cronbach’s α coefficient) of the HADS in this study was 0.855.

Investigators were trained to a uniform standard before formal commencement. Major investigators checked the integrity of daily questionnaires to ensure quality of investigation. The questionnaire was entered into Excel by two investigators on the same day to guarantee its accuracy.

### Statistical analysis

SPSS 25.0 was performed for data analysis. For non-normally continuous variables, described using median (IQR), and compared via the Mann–Whitney U test. Categorical variables were summarized as frequencies and percentages and analyzed via Chi-Squared test or Fisher’s exact test. Univariate and multivariate logistic regression were used to identify potential predictors. Initially, bivariate logistic models were performed to investigate a relationship between the Non-SI group and the SI group. Variables attained *p* < 0.05 were selected for further tests using a multivariate analysis model with a stepwise method. SI was allocated as a dependent variable, and other significant variables were entered as independent variables. Odds ratios (OR) and 95% confidence intervals (CI) were calculated as effect-size. Forest plots were used to visualize the results. Statistical significance was considered for the two-tailed tests at *p* < 0.05.

R 4.1.2 and “rms” and “ggplot2” packages were used to build a nomogram. Significant risk factors in multivariate analysis were selected to construct a prediction nomogram. In the nomogram, each variable has a separate predicted score, then adding them all up is the total score. Total predicted scores correspond to the predicted probabilities. Receiver operating characteristic (ROC) curve and the area under the curve (AUC) were calculated to evaluate discrimination accuracy of the nomogram. Based on the cutoff point, sensitivity and specificity were calculated. Bootstrap method and Hosmer–Lemeshow were performed to test internal validation and stability of fit. In addition, calibration plots and decision curve analysis (DCA) were performed to quantify the performance ability and clinical utility of the model.

## Results

### Participant characteristics

A total of 820 cancer patients were collected in the study, and the overall incidence of SI was 25.98%. 560 (338 males and 222 females) of them were assigned to the training set for nomogram construction and 260 (149 males and 111 females) were involved in the validation set. In the training set, the marital status (*p* = 0.002), medical financial burden (*p* < 0.001), living condition (*p* < 0.001), religious belief (*p* = 0.036), residence (*p* < 0.001), the level of demoralization (*p* < 0.001), depression (*p* < 0.001), anxiety (*p* < 0.001) and cancer staging (*p* < 0.001) were significant differences associated with SI between Non-SI group and SI group. Meanwhile, there was no statistical significance in age (*p* = 0.651), gender (*p* = 0.148), level of education (*p* = 0.920), income (*p* = 0.367), caretaker (*p* = 0.100), working status (*p* = 0.149), and type of cancer (*p* = 0.069). The baseline demographics of training database are shown in Table 1 and the demographic characteristics of validation database are shown in Additional file 1.

**Table 1:** Socio-demographic characteristics of training set (*N* = 560)

Variables	NSI, *N*(%)	SI, *N*(%)	^2^/Z	*P* value
Age, median (IQR), year	57(51,65)	57(50,65)	-0.453	0.651
Gender			2.098	0.148
Male	259(62.11%)	79(55.24%)		
Female	158(37.89%)	64(44.76%)		
Marital status			11.985	**0.002**
Married	398(95.44%)	125(87.41%)		
Spinsterhood	13(3.12%)	10(6.99%)		
Divorced or widowed	6(1.44%)	8(5.60%)		
Medical financial burden			28.528	** < 0.001**
Not at all	37(8.87%)	5(3.50%)		
A little	177(42.45%)	36(25.17%)		
Some	151(36.21%)	63(44.06%)		
Very much	52(12.47%)	39(27.27%)		
Living condition			13.885	** < 0.001**
Not live alone	399(95.68%)	124(86.71%)		
Live alone	18(4.32%)	19(13.29%)		
Religious belief			4.379	**0.036**
Yes	38(9.11%)	22(15.38%)		
No	379(90.89%)	121(84.62%)		
Residence			52.651	** < 0.001**
Rural	157(37.65%)	104(72.73%)		
Urban	260(62.35%)	39(27.27%)		
Level of education			0.929	0.920
Primary and below	145(34.77%)	51(35.66%)		
Junior high school diploma	139(33.33%)	43(30.07%)		
Senior high school diploma	96(23.02%)	36(25.18%)		
Some college	26(6.24%)	8(5.59%)		
Bachelors and advanced degree	11(2.64%)	5(3.50%)		
Income (yuan per month)			2.004	0.367
< 3000	66(15.83%)	28(19.58%)		
3000–5000	211(50.60%)	75(52.45%)		
≥ 5000	140(33.57%)	40(27.97%)		
Caretaker			6.262	0.100
Family member	368(88.25%)	123(86.01%)		
Nursing workers	3(0.72%)	5(3.50%)		
Friends	6(1.44%)	3(2.10%)		
Oneself	40(9.59%)	12(8.39%)		
Working status			2.085	0.149
Still working	144(34.53%)	59(41.26%)		
Sick rest	273(65.47%)	84(58.74%)		
Demoralization level^a^			64.732	** < 0.001**
Low demoralization	106(25.42%)	6(4.20%)		
Moderate demoralization	226(54.20%)	62(43.35%)		
High demoralization	85(20.38%)	75(52.45%)		
Depression level^b^			79.179	** < 0.001**
No	211(50.60%)	17(11.89%)		
Mild	119(28.54%)	50(34.96%)		
Moderate	83(19.90%)	71(49.65%)		
Severe	4(0.96%)	5(3.50%)		
Anxiety level^b^			52.082	** < 0.001**
No	159(38.13%)	20(13.98%)		
Mild	101(24.22%)	21(14.69%)		
Moderate	137(32.85%)	83(58.04%)		
Severe	20(4.80%)	19(13.29%)		
Cancer staging			57.373	** < 0.001**
I	82(19.66%)	4(2.80%)		
II	145(34.77%)	23(16.08%)		
III	111(26.62%)	63(44.06%)		
IV	79(18.95%)	53(37.06%)		
Cancer			21.219	0.069
Lung Cancer	141(33.81%)	34(23.77%)		
Colorectal Cancer	91(21.82%)	40(27.97%)		
Stomach Cancer	61(14.63%)	34(23.77%)		
Esophageal Cancer	20(4.80%)	3(2.10%)		
Liver Cancer	15(3.60%)	7(4.90%)		
Nasopharyngeal Cancer	11(2.64%)	1(0.70%)		
Bile duct cancer	10(2.40%)	2(1.40%)		
Lymphoma	7(1.68%)	1(0.70%)		
Thymus cancer	7(1.68%)	1(0.70%)		
Ovarian Cancer	3(0.72%)	4(2.80%)		
Pancreatic cancer	8(1.91%)	1(0.70%)		
Breast Cancer	6(1.44%)	2(1.40%)		
Cervical cancer	6(1.44%)	2(1.40%)		
Other cancer	31(7.43)	11(7.69%)		

SI: Suicidal ideation, scores of Self-rating Idea of Suicide Scale (SIOSS) ≥ 12

NSI: Non suicidal ideation, scores of self-rating Idea of Suicide Scale (SIOSS) < 12

^a^Measured with the Chinese version of Demoralization Scale II (DS-II-C)

^b^Measured with the The Hospital Anxiety and Depression Scale (HADS)

### Logistic regression variable screening results

**Fig. 1:**
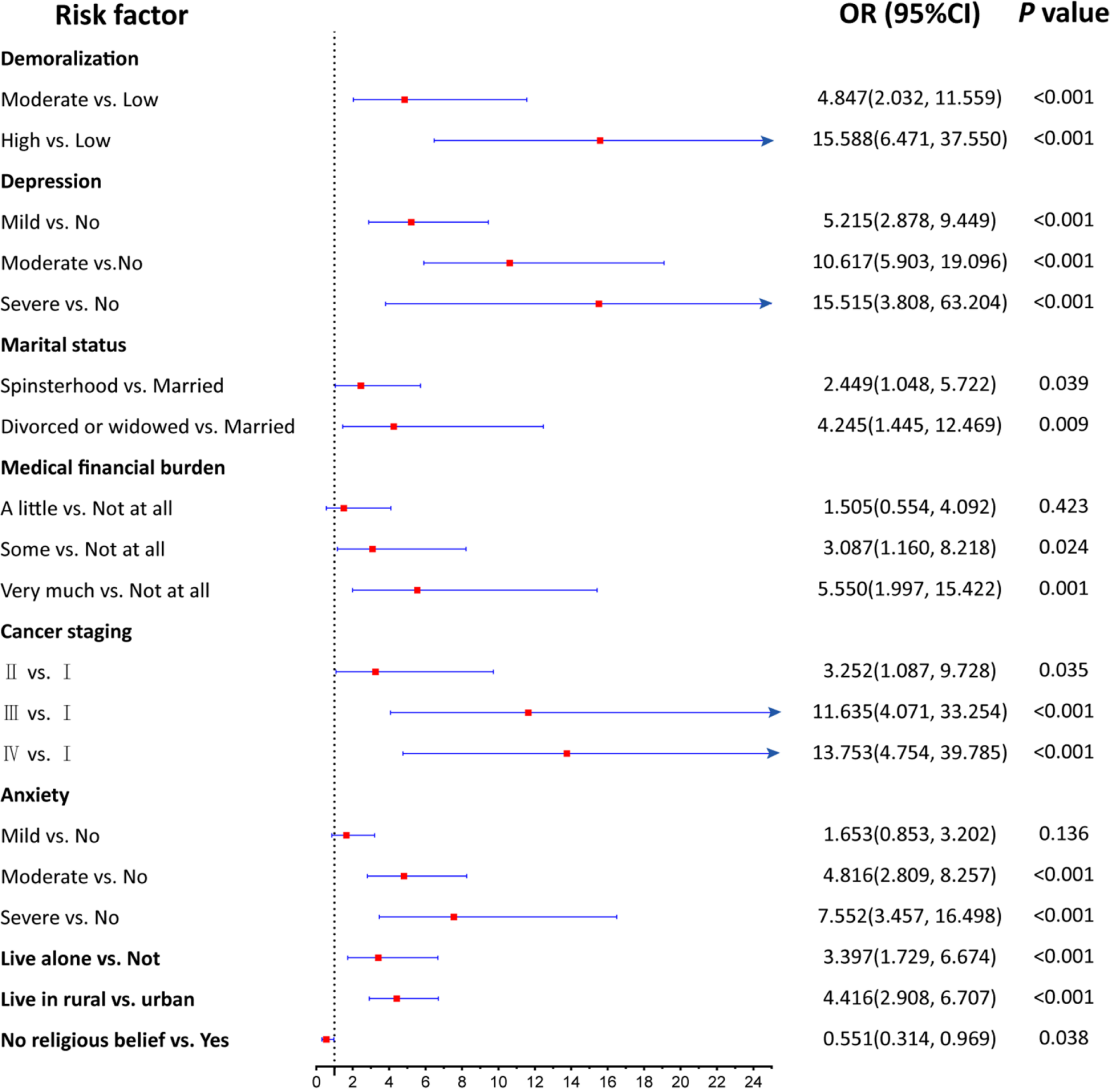
Odds Radio forest plot of univarible logistic regression

**Fig. 2:**
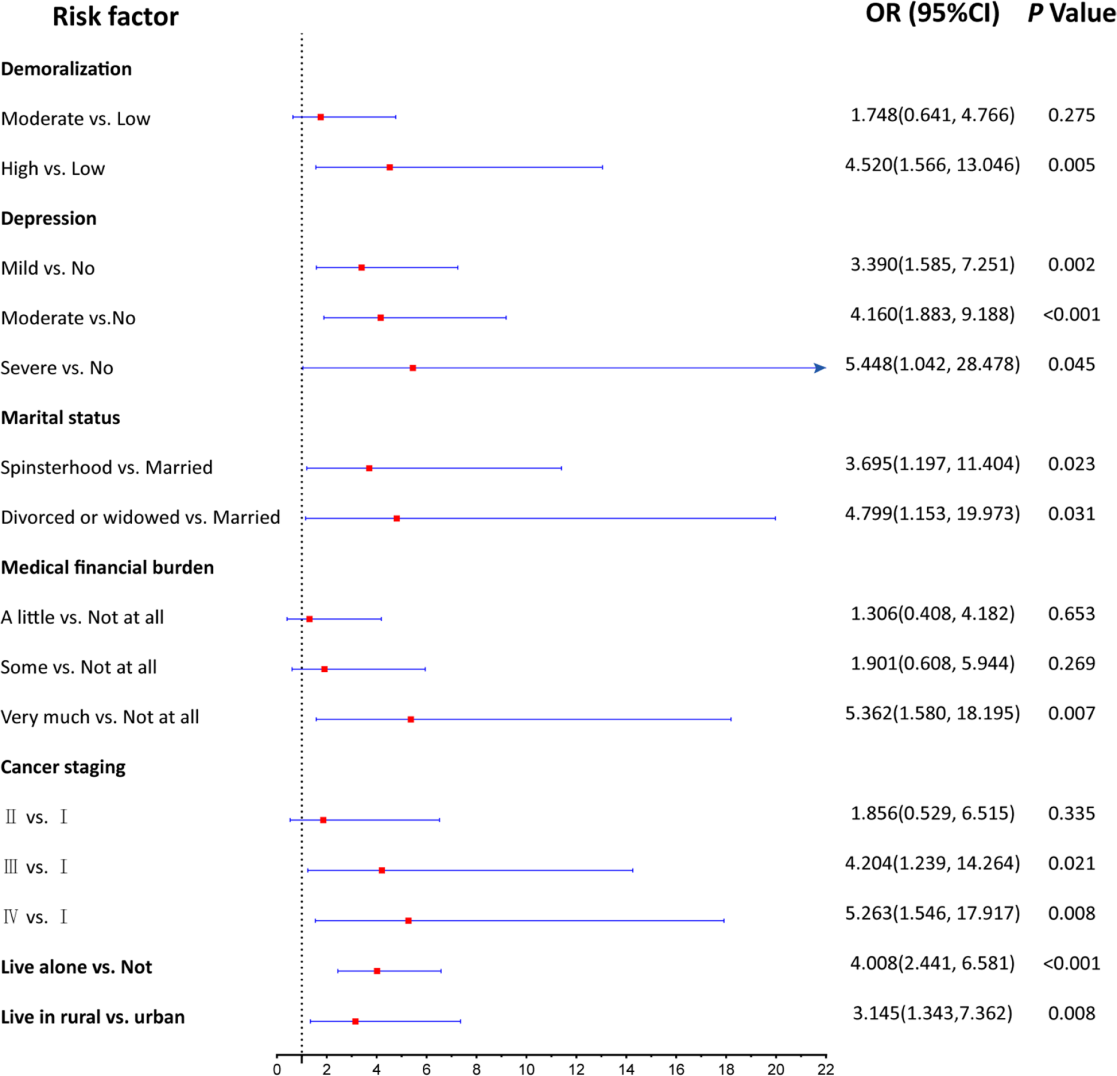
Odds Radio forest plot of multivarible logistic regression

Figure 1 and 2 were plotted to show the results of univariate and multivariate analyses. Figure 1 shows nine variables significantly related to SI through univariate analysis, and all of them were selected as potential predictors of the multivariate logistic regression. The multivariate analysis results (Fig. 2) showed that levels of demoralization and depression, marital status, medical financial burden, cancer staging, living condition and residence were independent influence factors (Specific values of these results are shown in Additional file 2).

### Nomogram construction and validation

**Fig. 3:**
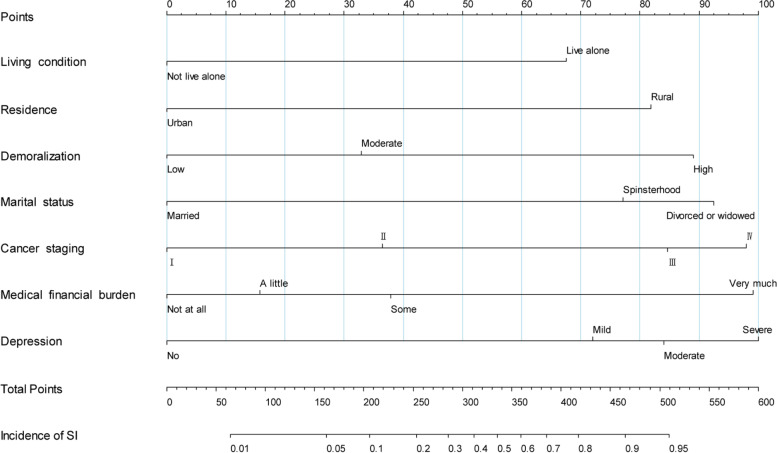
Nomogram for predicting suicidal ideation in cancer patients. For an individual patient, each variable corresponds to a single point at the top of nomogram (Points). The total points were summed up by all single points and are indicated in the second line from the bottom (Total Points), and each total point corresponds to a probability of suicidal ideation

The predictive nomogram of SI was constructed by combining the above independent prediction variables, which were analyzed by multiple logistic regression (Fig. 3). All predictive variables were projected to obtain the matching points on the ruler at the top then added to obtain the total points, with a corresponding prediction probability below. The higher the total score, the greater the likelihood of SI.

**Fig. 4:**
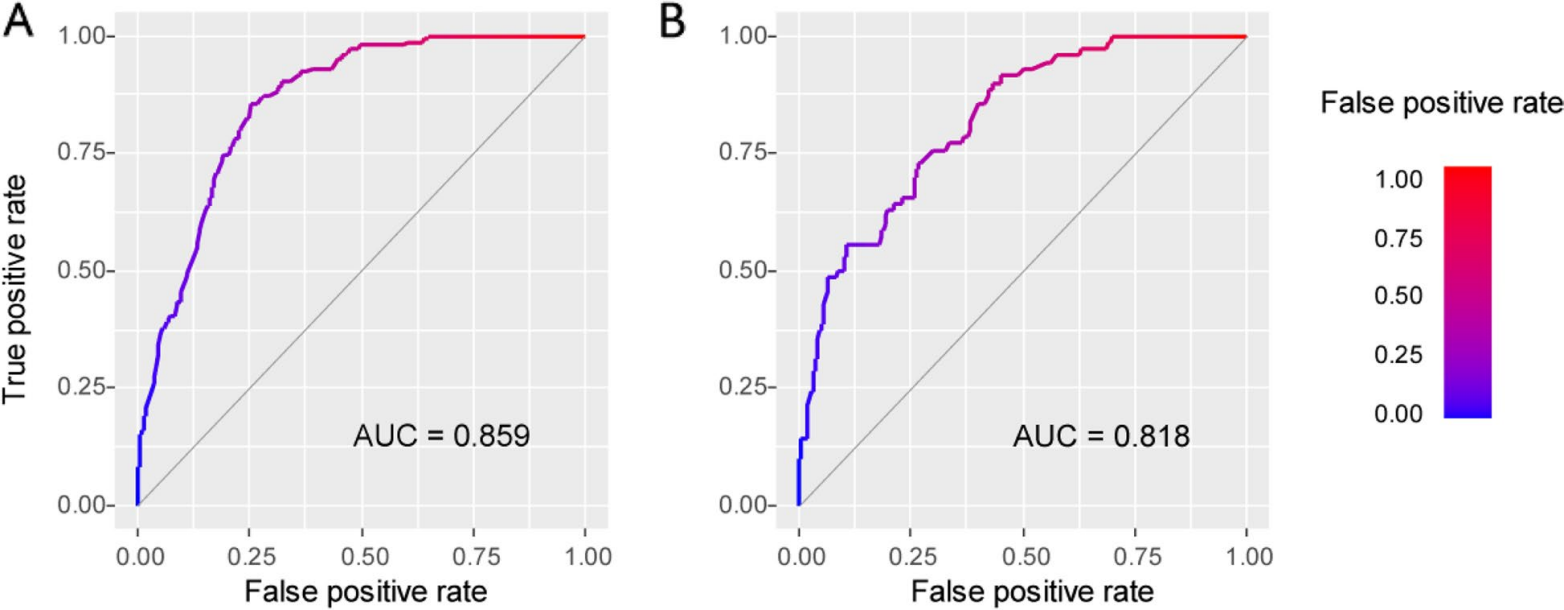
ROC curves of nomogram for predicting the probability of SI in training set (**A**) and validation set (**B**). The horizontal axis means the false positive rate of the risk prediction. The vertical axis means the true positive rate of the risk prediction. The color line represents the performance of nomogram. ROC: Receiver operating characteristic curve; AUC: Area under curve; SI: Suicidal ideation

We conducted internal and external validation of this nomogram. Collectively, these results indicate that the nomogram is a reliable tool for predicting SI in cancer patients. The Hosmer–Lemeshow test for training set was 0.410. AUC of the nomogram were 0.859 (95% CI: 0.827–0.890) and 0.818 (95% CI: 0.764–0.873) in the training and validation sets, respectively (Fig. 4). In the training set, the Youden index was 0.601, the cutoff value was 0.216, and the sensitivity and specificity were 0.853 and 0.748, respectively.

**Fig. 5:**
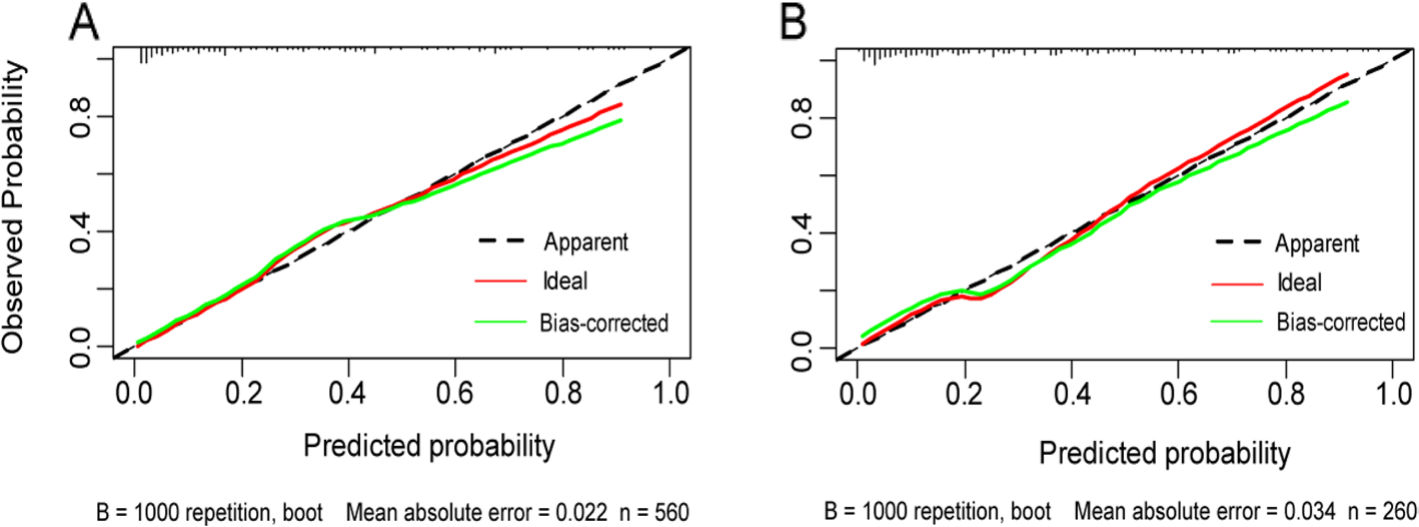
Calibration curves of nomogram for predicting the probability of SI in training set (**A**) and validation set (**B**). Internal validation of the nomogram was performed using a corrected calibration curve within 1000 bootstrap samples. The horizontal axis represents the predicted probability of SI. The vertical axis represents the actual SI probability. The diagonal dotted line represents a perfect prediction of an ideal model. The green line represents the performance of the nomogram, of which a closer fit to the diagonal dotted line represents a better prediction. SI: Suicidal ideation.

**Fig. 6:**
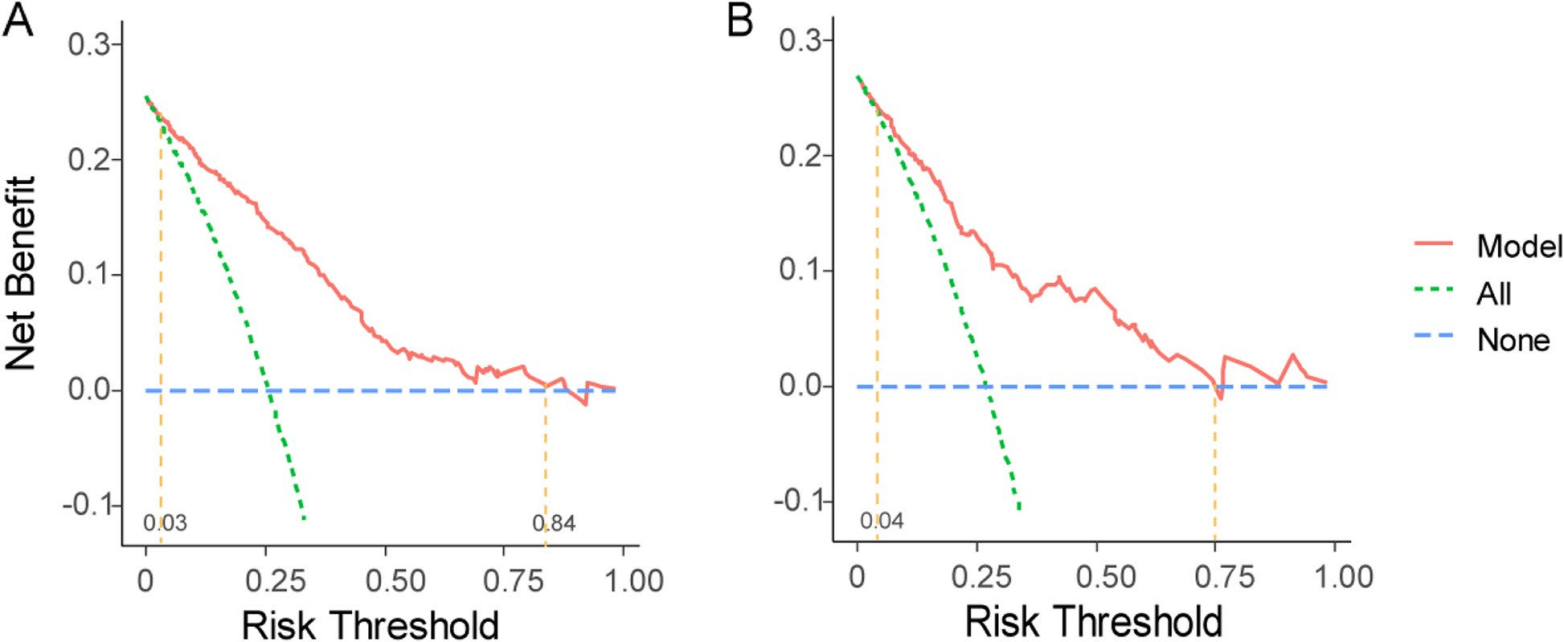
DCA curves of nomogram for predicting the probability of SI in training set (**A**) and validation set (**B**). The horizontal and vertical axes represent the threshold probability and net benefit, respectively. The lines between the horizontal axis and vertical axis display the benefit of different predictive variables. The DCA curves show that if the threshold probability is 3–84%, using this nomogram in the current study to predict SI risk could add more benefit. DCA: Decision curve analysis; SI: Suicidal ideation

**Fig. 7:**
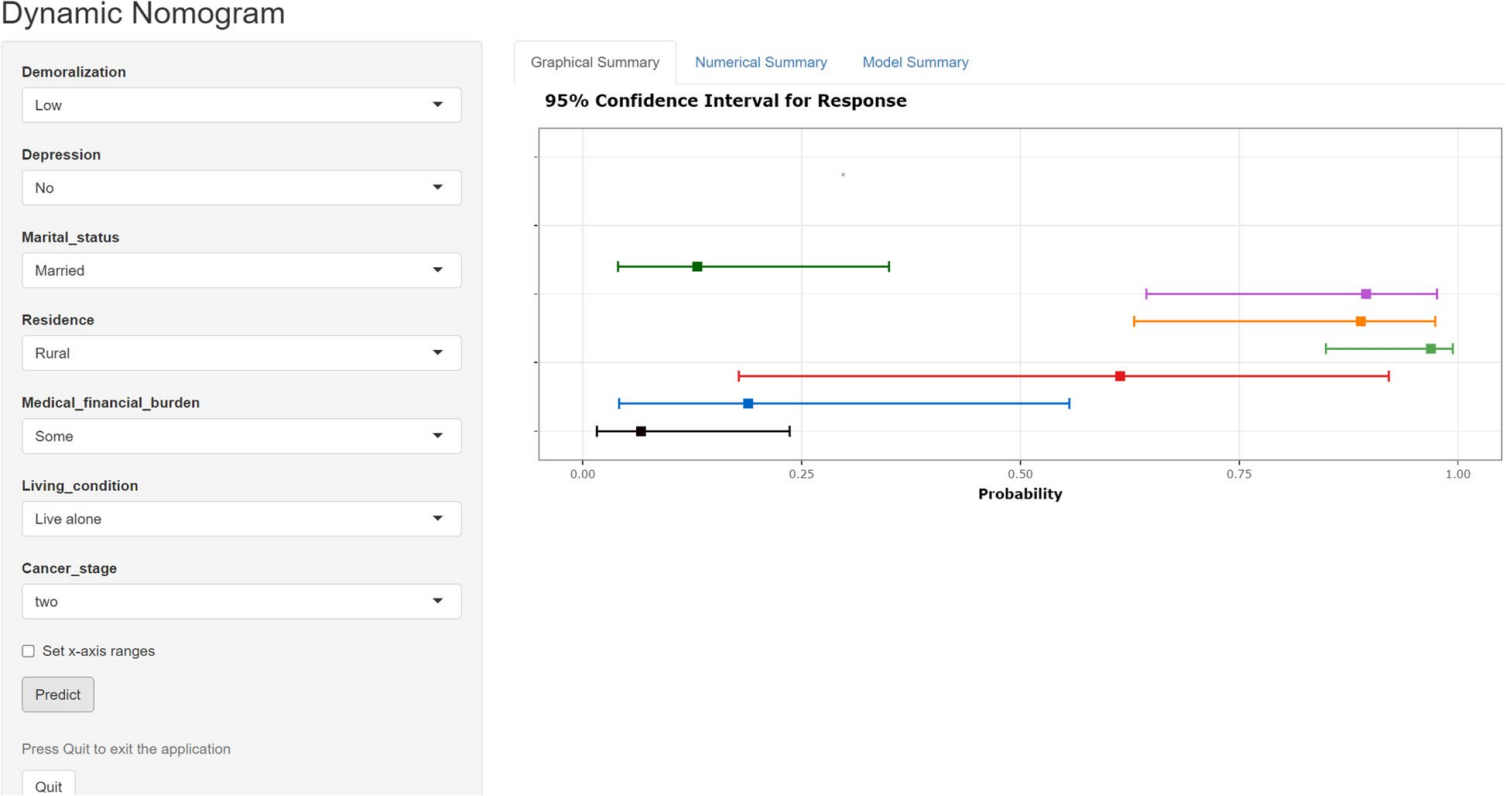
The online nomogram for predicting suicidal ideation in cancer patients. Clinical medical workers could choose options from drop-down menus based on the actual situation of patients. After determining the parameters, the probability of suicidal ideation can be obtained by clicking the “predict” button. The “Graphical Summary” shows probability of prediction and 95% confidence interval, and the mouse over the square show specific parameters. In addition, the “Numerical Summary” and “Model Summary” describe the specific prediction results and model parameters. The online nomogram for predicting suicidal ideation in cancer patients is available at https://si-nomogram.shinyapps.io/dynnomapp/

The calibration curve was plotted in Fig. 5. We adopted the bootstrap method to form the curves, which was repeated 1000 times, and results showed the bias corrected curve and apparent curve were both similar with reference line, demonstrating good agreement between predicted and observed risk of SI. In addition, we plotted DCA curve to evaluate clinical benefits of the nomogram. Results of DCA curve showed model yields net benefit to a wide range of approximately 3% to 84% in training set and 4% to 75% in validation set, which means model is beneficial in making decisions in clinical settings (Fig. 6). For clinical convenience, we uploaded an online program using the “DynNom” package of R (https://cran.r-project.org/web/packages/DynNom/index.html); it is available at https://si-nomogram.shinyapps.io/dynnomapp/. The probability of SI can be obtained by clicking the “predict” button, after the parameters have been determined (Fig. 7).

## Discussion

This study indicated that 25.98% cancer patients generated suicidal ideation, and the risk factors for SI are demoralization, depression, advanced cancer staging, medical financial burden, single status, living in rural areas, and living alone. In terms of overall incidence, the results were consistent with previous studies, which showed a higher probability of SI among cancer patients compared with the general population [29, 30]. One possible explanation may be that heavy financial pressure, poor prognosis, fragile psychology and torture of treatment make cancer patients so overwhelmed that they have to think about death. Another possible reason is that cancer patients are getting younger. Young cancer patients are under a lot of stress from both family and society but have less resilience to withstand, so they are more likely to choose the extreme method. Our results also showed that the incidence of SI was lower in patients with early cancer staging than in patients with advanced cancer staging. Advanced cancer staging often predicts a poor prognosis and shorter survival time. At the same time, patients with advanced cancer staging often have metastases and are in severe disease states, so they may suffer more physical and mental predicament, and as a result increased the risk of SI.

History of mental illness was the strongest risk factor associated with SI [31]. Our study finding that demoralization and depression could accelerate SI in cancer patients, which is consistent with Wu and Sun [27, 32]. Although the clinical manifestations of demoralization and depression are similar, there are differences between the two diagnoses. Depression is a physiological factor and can be regulated by antidepressant drugs, while demoralization is a subjective incompetence, and drugs may not improve the situation of demoralization. Patients with demoralization could not be comprehensively recognized because they were ignored by clinical staff, and thus had higher SI. Cancer patients suffer more pain than any other disease [33, 34]. Cancer diagnosis, torment of disease, as well as treatment side effects, all of which can lead to hopelessness, demoralization and further generate SI. Anxiety patients may have elevated risk of SI [35]. However, a surprising finding in this present study is that anxiety was no longer a significant influence on SI after controlling for other variables. This is probably because anxiety patients are thinking about how to regain health more than suicide, and medications could also reduce anxiety levels. Otherwise, patients might turn anxiety into depression or demoralization if they were hurt for a long time, therefore anxiety levels were not significant in the study. This finding should also be a wake-up call to medical staff, highlighting the vulnerable subgroups of cancer patients with demoralization who may have a higher probability of SI. Screening for demoralization should also be part of admission screening for cancer patients.

Considerable evidence has confirmed that marital status was a significant factor for SI in cancer patients [36, 37]. Our study provides additional support for the marriage support hypothesis. We discovered that marriage was an independent protective factor for SI, even after adjusting for confounding factors, and that spouses were more supportive than other relatives. Physically, the spouse is more aware of the patient's needs, and emotionally, the spouse is better understanding the patient's preferences. In addition, married patients may better adhere to therapies with spousal encouragement and companionship, which also contributes to an increased overall treatment trajectory resulting in improved prognosis. As briefly mentioned before, the findings suggest companionship does reduce SI in patients. In our research, SI appears to be more prevalent among solitary patients, and the common rationale might be that patients who live alone feel more social isolation and loneliness [38]. It also suggests that more psychosocial resources should be integrated into the care of patients who live alone.

The effects of the financial burden on cancer patients are evident [39, 40]. Cancer testing and treatments are the key to treatment for cancer patients, but may subject the patient to extreme medical financial distress or burden. To maintain continuity of treatment, cancer patients have to reduce their original living expenses or even borrow money embarrassingly for treatment. Thus, a double whammy of illness and spirits may further intensify the promotion of SI. Simultaneously, our results indicate that cancer patients living in rural areas were more likely to generate SI. In addition to monetary reasons, there are also healthcare access and quality inequities in urban and rural areas. Urban means better treatments, more experienced doctors, and higher quality medical facility. The large gap between urban and rural areas points to the need for reform, as access to mental health services should be included in the rural public health scheme.

Cancer patients’ SI is a huge but largely preventable public health concern [41]. Identifying high risk patients is the first step to suicide prevention and also a recommended preventive strategy [42, 43]. Only if patients with a higher probability of SI could be identified and then psychosocial interventions could be applied to them in advance. But in fact, the majority of these studies focused on the incidence of SI among psychiatric patients, students, maternal or suicide outcomes in databases, rather than the occurrence of individual SI. Also, most medical staff did not measure SI because the questions and questionnaires about SI may have negative effects on patients. Few studies have built predictive models for SI of cancer patients, but they did not include emotional factors, the most important factor in SI, and lack of clinical usefulness [44, 45]. While our model also shows favorable discrimination performance and clinical utility. Medical staff could better predict SI risk based on the different characteristics of cancer patients using the guided nomogram, which offered an effective clinical predictive model and made early identification possible.

### Strengths and limitations

To our knowledge, this is the first study to construct an electronic nomogram for predicting SI in cancer patients. This nomogram includes both sociodemographic and psychological characteristics, so that it can identify high risk patients who need to be closely observed more accurately. Furthermore, the effective and easily applied nomogram rarely includes provocative or negative information, so it is less harmful to patients than conventional screening scales. The results are encouraging and provide the first step towards using screening directly in clinical practice, which will help medical staff take early steps to reduce suicide mortality rates among cancer patients.

Inevitably, our study exhibits the following limitations. First of all, our conclusions should not be extended to children and teenage cancer patients, because this study focused on adult cancer patients. Additionally, this study is a cross-sectional study, so firm casual conclusions may not be drawn. However, the findings were still valuable in providing a preliminary screening tool for SI in cancer patients. Lastly, we only included patients who were willing to participate in our survey, while other patients who refused to be tested probably had existing SI. Therefore, the incidence of SI may be underestimated.

## Conclusion

Our findings demonstrate suicidal ideation is still a critical issue in cancer patients. Cancer patients with demoralization, depression and advanced cancer staging were identified as high-risk group at suicidal ideation. Therefore, screening and attention should be focused on these patients.

## Supplementary Information

**Additional file 1.** Socio-demographiccharacteristics of training dataset (*N*=560) and validation dataset (*N*=260).

**Additional file 2.** Univariate and multivariate regression analysesfor suicidal ideation (SI) in cancer patients (*N*=560).

## Data Availability

The datasets used and/or analyzed during the current study are available from the corresponding author on reasonable request.

## Abbreviations

IQR: Interquartile Range
SI: Suicidal Ideation
SIOSS: Self-rating Idea of Suicide Scale
HADS: Self-rating Idea of Suicide Scale
ROC: Receiver Operating Characteristic Curve
AUC: Area Under the Curve
DCA: Decision Curve Analysis
WHO: World Health Organization
ICD-10: International Classification of Diseases-10 criteria
