# Supplementary material for: Risk factor analysis and nomogram construction for predicting suicidal ideation in patients with cancer
Source: BMC Psychiatry. 2022 May 24;22:353. doi: 10.1186/s12888-022-03987-z (PMC9128228; doi:10.1186/s12888-022-03987-z)
Supplement: Supplementary file 1 — Additional file 1. Socio-demographiccharacteristics of training dataset (N=560) and validation dataset (N=260). [file 12888_2022_3987_MOESM1_ESM.doc]

**Additional file** **1** Socio-demographic characteristics of training set (*N*=560) and validation set (*N*=260)

| **Variables** | **Training set****, *N*(%)** | **Validation set, *N*(%)** | **χ^2^/Z** | ***P* value** |
| --- | --- | --- | --- | --- |
| Age, median (IQR), year | 57(50, 65) | 58(50,65) | -0.892 | 0.372 |
| Gender |  |  |  |  |
| Male | 338(60.36%) | 149(57.31%) | 0.685 | 0.408 |
| Female | 222(39.64%) | 111(42.69%) |  |  |
| Marital status |  |  | 0.973 | 0.615 |
| Married | 523(93.39%) | 238(91.54%) |  |  |
| Spinsterhood | 23(4.11%) | 13(5.00%) |  |  |
| Divorced or widowed | 14(2.50%) | 9(3.46%) |  |  |
| Medical financial burden |  |  | 4.947 | 0.176 |
| Not at all | 42(7.50%) | 11(4.23%) |  |  |
| A little | 213(38.04%) | 115(44.23%) |  |  |
| Some | 214(38.21%) | 95(36.54%) |  |  |
| Very much | 91(16.25%) | 39(15.00%) |  |  |
| Living condition |  |  | 1.775 | 0.183 |
| Not live alone | 523(93.39%) | 236(90.77%) |  |  |
| Live alone | 37(6.61%) | 24(9.23%) |  |  |
| Religious belief |  |  | 1.308 | 0.253 |
| Yes | 60(10.71%) | 35(13.46%) |  |  |
| No | 500(89.29%) | 225(86.54%) |  |  |
| Residence |  |  | 0.644 | 0.422 |
| Rural | 261(46.61%) | 129(49.62%) |  |  |
| Urban | 299(53.39%) | 131(50.38%) |  |  |
| Level of education |  |  | 5.621 | 0.229 |
| Primary and below | 196(35.00%) | 87(33.46%) |  |  |
| [Junior](javascript:;) [high](javascript:;) [school](javascript:;) diploma | 182(32.50%) | 78(30.00%) |  |  |
| [Senior](javascript:;) [high](javascript:;) [school](javascript:;) diploma | 132(23.57%) | 61(23.46%) |  |  |
| Some [college](javascript:;) | 34(6.07%) | 18(6.92%) |  |  |
| Bachelors and advanced degree | 16(2.86%) | 16(6.16%) |  |  |
| Income (yuan per month) |  |  | 1.225 | 0.542 |
| ＜3000 | 94(16.79%) | 47(18.08%) |  |  |
| 3000-5000 | 286(51.07%) | 122(46.92%) |  |  |
| ≥5000 | 180(32.14%) | 91(35.00%) |  |  |
| Caretaker |  |  | 3.249 | 0.355 |
| Family member | 491(87.68%) | 238(91.54%) |  |  |
| Nursing workers | 8(1.43%) | 4(1.54%) |  |  |
| Friends | 9(1.61%) | 3(1.15%) |  |  |
| Oneself | 52(9.28%) | 15(5.77%) |  |  |
| Working state |  |  | 2.761 | 0.097 |
| Still working | 203(36.25%) | 110(42.31%) |  |  |
| Sick rest | 357(63.75%) | 150(57.69%) |  |  |
| Cancer staging |  |  | 0.435 | 0.933 |
| Ⅰ | 86(15.36%) | 36(13.84%) |  |  |
| Ⅱ | 168(30.00%) | 77(29.62%) |  |  |
| Ⅲ | 174(31.07%) | 85(32.69%) |  |  |
| Ⅳ | 132(23.57%) | 62(23.85%) |  |  |
| Cancer |  |  | 10.469 | 0.655 |
| Lung Cancer | 175(31.25%) | 84(32.31%) |  |  |
| Colorectal Cancer | 131(23.39%) | 63(24.23%) |  |  |
| Stomach Cancer | 95(16.96%) | 34(13.08%) |  |  |
| Esophageal Cancer | 23(4.11%) | 17(6.54%) |  |  |
| Liver Cancer | 22(3.93%) | 8(3.08%) |  |  |
| Nasopharyngeal Cancer | 12(2.14%) | 6(2.31%) |  |  |
| Bile duct cancer | 12(2.14%) | 7(2.69%) |  |  |
| Lymphoma | 8(1.43%) | 5(1.92%) |  |  |
| Thymus cancer | 8(1.43%) | 6(2.31%) |  |  |
| Ovarian Cancer | 7(1.25%) | 5(1.92%) |  |  |
| [Pancreatic](javascript:;) [cancer](javascript:;) | 9(1.61%) | 3(1.15%) |  |  |
| Breast Cancer | 8(1.43%) | 6(2.31%) |  |  |
| Cervical cancer | 8(1.43%) | 5(1.92%) |  |  |
| Other cancer | 42(7.50%) | 11(4.23%) |  |  |
