# Supplementary material for: Risk factor analysis and nomogram construction for predicting suicidal ideation in patients with cancer
Source: BMC Psychiatry. 2022 May 24;22:353. doi: 10.1186/s12888-022-03987-z (PMC9128228; doi:10.1186/s12888-022-03987-z)
Supplement: Supplementary file 2 — Additional file 2. Univariate and multivariate regression analysesfor suicidal ideation (SI) in cancer patients (N=560). [file 12888_2022_3987_MOESM2_ESM.doc]

**Additional file 2** Univariate and multivariate logistic regression analyse for suicidal ideation (SI) in cancer patients (*N*=560)

| **Variables** | **Univariate** **analyses** | | |  | **Multivariate analyses** | | |
| --- | --- | --- | --- | --- | --- | --- | --- |
|  | ***β*** | **OR (95% CI)** | ***P* value** |  | ***β*** | **OR (95% CI)** | ***P* value** |
| Demoralization level ^a^ |  |  |  |  |  |  |  |
| Low | Ref |  |  |  | Ref |  |  |
| Moderate | 1.578 | 4.847(2.032,11.559) | <0.001 |  | 0.558 | 1.748(0.641,4.766) | 0.275 |
| High | 2.747 | 15.588(6.471,37.550) | <0.001 |  | 1.508 | 4.520(1.566,13.046) | 0.005 |
| Depression level ^b^ |  |  |  |  |  |  |  |
| No | Ref |  |  |  | Ref |  |  |
| Mild | 1.652 | 5.215(2.878,9.449) | <0.001 |  | 1.221 | 3.390(1.585,7.251) | 0.002 |
| Moderate | 2.362 | 10.617(5.903,19.096) | <0.001 |  | 1.425 | 4.160(1.883,9.188) | <0.001 |
| Severe | 2.742 | 15.515(3.808,63.204) | <0.001 |  | 1.695 | 5.448(1.042,28.478) | 0.045 |
| Marital status |  |  |  |  |  |  |  |
| Married | Ref |  |  |  | Ref |  |  |
| Spinsterhood | 0.896 | 2.449(1.048,5.722) | 0.039 |  | 1.307 | 3.695(1.197,11.404) | 0.023 |
| Divorced or widowed | 1.446 | 4.245(1.445,12.469) | 0.009 |  | 1.568 | 4.799(1.153,19.973) | 0.031 |
| Medical financial burden |  |  |  |  |  |  |  |
| Not at all | Ref |  |  |  | Ref |  |  |
| A little | 0.409 | 1.505(0.554,4.092) | 0.423 |  | 0.267 | 1.306(0.408,4.182) | 0.653 |
| Some | 1.127 | 3.087(1.160,8.218) | 0.024 |  | 0.642 | 1.901(0.608,5.944) | 0.269 |
| Very much | 1.714 | 5.550(1.997,15.422) | 0.001 |  | 1.679 | 5.362(1.580,18.195) | 0.007 |
| Cancer staging |  |  |  |  |  |  |  |
| Ⅰ | Ref |  |  |  | Ref |  |  |
| Ⅱ | 1.179 | 3.252(1.087,9.728) | 0.035 |  | 0.618 | 1.856(0.529,6.515) | 0.335 |
| Ⅲ | 2.454 | 11.635(4.071,33.254) | <0.001 |  | 1.436 | 4.204(1.239,14.264) | 0.021 |
| Ⅳ | 2.621 | 13.753(4.754,39.785) | <0.001 |  | 1.661 | 5.263(1.546,17.917) | 0.008 |
| Living condition |  |  |  |  |  |  |  |
| Not live alone | Ref |  |  |  | Ref |  |  |
| Live alone | 1.223 | 3.397(1.729,6.674) | <0.001 |  | 1.388 | 4.008(2.441,6.581) | <0.001 |
| Residence |  |  |  |  |  |  |  |
| Urban | Ref |  |  |  | Ref |  |  |
| Rural | 1.485 | 4.416(2.908,6.707) | <0.001 |  | 1.146 | 3.145(1.343,7.362) | 0.008 |
| Religious belief |  |  |  |  |  |  |  |
| Yes | Ref |  |  |  |  |  |  |
| No | -0.595 | 0.551(0.314,0.969) | 0.038 |  |  |  |  |
| Anxiety level ^b^ |  |  |  |  |  |  |  |
| No | Ref |  |  |  |  |  |  |
| Mild | 0.503 | 1.653(0.853,3.202) | 0.136 |  |  |  |  |
| Moderate | 1.572 | 4.816(2.809,8.257) | <0.001 |  |  |  |  |
| Severe | 2.022 | 7.552(3.457,16.498) | <0.001 |  |  |  |  |
| Working state |  |  |  |  |  |  |  |
| Still working | Ref |  |  |  |  |  |  |
| Sick rest | -0.286 | 0.751(0.509,1.108) | 0.149 |  |  |  |  |
| Gender |  |  |  |  |  |  |  |
| Male | Ref |  |  |  |  |  |  |
| Female | 0.284 | 1.328(0.904,1.951) | 0.148 |  |  |  |  |
| Level of education |  |  |  |  |  |  |  |
| Primary and below | Ref |  |  |  |  |  |  |
| [Junior](javascript:;) [high](javascript:;) [school](javascript:;) diploma | -0.128 | 0.880(0.551,1.404) | 0.591 |  |  |  |  |
| [Senior](javascript:;) [high](javascript:;) [school](javascript:;) diploma | 0.064 | 1.066(0.648,1.755) | 0.801 |  |  |  |  |
| Some [college](javascript:;) | -0.134 | 0.875(0.372,2.055) | 0.759 |  |  |  |  |
| Bachelors and advanced degree | 0.256 | 1.292(0.428,3.899) | 0.649 |  |  |  |  |
| Income(yuan per month) |  |  |  |  |  |  |  |
| ＜3000 | Ref |  |  |  |  |  |  |
| 3000-5000 | -0.177 | 0.838(0.501,1.402) | 0.500 |  |  |  |  |
| ≥5000 | -0.395 | 0.673(0.383,1.185) | 0.170 |  |  |  |  |
| Caretaker |  |  |  |  |  |  |  |
| Family member | Ref |  |  |  |  |  |  |
| Nursing workers | 1.607 | 4.986(1.175,21.170) | 0.029 |  |  |  |  |
| Friends | 0.403 | 1.496(0.369,6.072) | 0.573 |  |  |  |  |
| Oneself | -0.108 | 0.898(0.456,1.766) | 0.754 |  |  |  |  |

^a^ Measured with the Chinese version of Demoralization Scale II (DS-Ⅱ-C).

^b^ Measured with the The Hospital Anxiety and Depression Scale (HADS).
